# The application of transcriptional blood signatures to enhance our understanding of the host response to infection: the example of tuberculosis

**DOI:** 10.1098/rstb.2013.0427

**Published:** 2014-06-19

**Authors:** Simon Blankley, Matthew Paul Reddoch Berry, Christine M. Graham, Chloe I. Bloom, Marc Lipman, Anne O'Garra

**Affiliations:** 1 Division of Immunoregulation, MRC National Institute for Medical Research, London NW7 1AA, UK; 2 Department of Respiratory Medicine, Imperial College Healthcare NHS Trust, St Mary's Hospital, London W2 1NY, UK; 3 NHLI, Faculty of Medicine, Imperial College London, London, UK; 4 Department of Respiratory Medicine, Royal Free London NHS Foundation Trust, London, UK; 5 Division of Medicine, University College London, London, UK

**Keywords:** immune response, infection, tuberculosis

## Abstract

Despite advances in antimicrobials, vaccination and public health measures, infectious diseases remain a leading cause of morbidity and mortality worldwide. With the increase in antimicrobial resistance and the emergence of new pathogens, there remains a need for new and more accurate diagnostics, the ability to monitor adequate treatment response as well as the ability to predict prognosis for an individual. Transcriptional approaches using blood signatures have enabled a better understanding of the host response to diseases, leading not only to new avenues of basic research, but also to the identification of potential biomarkers for use in diagnosis, prognosis and treatment monitoring.

## 1. Introduction

Since the 1990s, whole genome expression mRNA microarray technology has been available as a tool to researchers. It involves profiling the expression levels of thousands of genes simultaneously and is increasingly being used to advance our understanding of the complex transcriptional response that occurs as a consequence of a disease process [1–3]. Modern microarray platforms are capable of reliably and reproducibly measuring the expression of over 40 000 mRNA transcripts, which can encompass all of the known functional human genome. By comparing between cohorts of individuals, or by sequentially sampling over a time course, it is possible to delineate a comparative transcriptional response to a given perturbation. Depending on the scale of the response, many hundreds or thousands of significantly differentially regulated genes may be identified. To comprehend the biological relevance of these complex data, bioinformatics tools have been developed that use known relationships between genes and their biological functions. With these tools, it is possible to determine the most biologically significant groups of differentially regulated genes as well as key transcriptional regulatory pathways/networks perturbed upon a given stimulus/disease [4–7].

## 2. Gene expression profiling using peripheral blood

The early gene expression studies involving human peripheral blood focused on cancer and autoimmune conditions [2,8]. The use of peripheral blood for whole genome expression studies in disease has a number of advantages. Peripheral blood is easily accessible, whereas the site of primary infection in certain diseases may not be easy to access, and it is possible to obtain reproducible blood transcriptional profiles from volumes as small as that obtained from a finger prick [9–11]. Blood represents a reservoir where there is a dynamic exchange of chemokines, cytokines and cells trafficking to and from sites of active disease and the lymphatic system. These cells include neutrophils, basophils, eosinophils, T cells and B cells; however, the cellular composition of the blood can vary depending on the ethnic background [12] of the patient as well as the scale and specificity of the host response to a disease. Different immune cells may have different baseline levels of gene expression as well as different transcriptional amplification programmes [13,14]. The interpretation of blood-derived transcriptional signatures therefore has to be made in the context of the cellular composition of the blood being sampled. Analysis of a subset of cells such as peripheral blood mononuclear cells (PBMCs) has been used as an attempt to control for this variable, although this excludes from analysis granulocytes such as neutrophils, which make up the greatest proportion of the immune cells found within blood and are important innate immune system effector cells. Furthermore, neutrophils have been seen to be important drivers of the blood transcriptional signature in certain diseases [15,16]. The superiority or suitability of PBMCs or whole blood leucocytes (globin RNA reduced) for transcriptional signatures in disease has yet to be rigorously compared. In addition, expression analysis of the subsets of blood leucocytes has also been an informative approach [14–17]. In some cases, this approach may be necessary to reveal signatures expressed in a discrete cell population that is present only in low numbers in the peripheral blood [18], or where the sensitivity of specific gene expression profiles may be masked if a unique profile is present in the cells found in low frequency in human blood [19]. An alternative approach is to apply computational deconvolution methods; this generates data for both the heterogeneous sample (whole blood, PBMCs) and its specific cell subsets [20].

During inflammatory, autoimmune diseases, cancers or infectious diseases the changes observed in a transcriptional blood signature can represent changes in cell numbers, which may be reduced as a consequence of apoptosis or migration of cells from the blood to other tissues. Conversely, increased cell numbers may result from proliferation of cells or migration of cells from the tissues or bone marrow into the blood. Alternatively, the transcriptional changes may be due to discrete changes in transcription within a specific cellular population as a consequence of perturbations resulting from the disease state. As an example, early in the host response to a pathogen, pattern recognition receptors, which are expressed on a wide variety of cell types including cells of the innate immune system, recognize the highly conserved microbial products of pathogens [21]. Binding of these receptors results in activation of signalling pathways and via transcriptional regulators the induction of transcriptional programmes [22]. Different pattern recognition receptors can recognize different microbial products and activate specific transcriptional programmes, enabling a transcriptional response appropriate to the pathogen. These directly activated immune cells can traffic from the site of infection and enter the blood stream either directly or via the lymphatic system. Overall, therefore, the transcriptional signatures observed in the blood in response to a disease may be as a consequence of changes in absolute cell numbers, changes in the proportions of cell types as well as changes in transcription within cell populations.

The specificity of a transcriptional signature for a particular disease may lie in the combination of activated pathways and transcription programmes rather than in uniquely activated disease-specific genes. Tools have been designed to characterize this transcriptional response, such as gene set enrichment analysis, transcriptional networks and modular approaches that can identify combinations of transcriptional programmes associated with diseases [23]. Algorithmic approaches such as the molecular distance to health or variations on this principle have used gene expression data to assess severity of disease or quantify disease response to treatment [15,24,25].

## 3. Infectious diseases

Gene expression studies in infectious diseases have been used to identify transcriptional signatures that differentiate between bacterial and viral infections [14,26,27], bacterial meningitis [28], acute febrile [29] and viral illnesses [30,31], as well as specific disease pathogens such as *Burkholderia* *pseudomallei* [24,32], dengue virus [33–35], human immunodeficiency virus [36,37], *Mycobacterium leprae* [38], *Staphylococcus aureus* [39], *Streptococcus pneumoniae* [40], *Salmonella enterica* [41] and *Mycobacterium tuberculosis* [15–17,25,42–49].

One of the challenges in infectious diseases is to identify the causative agent early in the disease, so that appropriate therapy can be initiated early or inappropriate therapy avoided. The traditional approach involves identification of the pathogen itself either by direct visualization, culture, nucleic acid amplification of pathogen-specific DNA or by measuring a specific antibody-mediated response. These approaches are not feasible for all infectious diseases, owing to either the inaccessibility of the tissue or difficulty in culturing the pathogen, or because the generation of an antibody-specific response is too slow to guide initial therapy. An ability to identify a transcriptional signature specific for an infectious disease based on the early host response would, therefore, be advantageous for use in diagnosis as well as revealing information about the early host response. Studies have shown that host blood transcriptional signatures can differentiate between different infectious diseases [14,27,29]. These signatures reveal that the broad response to viral infections includes well recognized, upregulated viral response elements including interferon-inducible genes [14,27], and can discriminate not only between bacterial and viral infection [27] but also between different viral infections [14,30].

Gene transcription profiling during early infectious disease has been used to identify biomarkers that can potentially be used to predict outcome as well as give a better understanding of the potential deficiencies or exaggerated transcriptional responses that correlate with poor outcome. In HIV, pathways involved in apoptosis, development, cell cycle and DNA damage were found to be differentially regulated in patients with rapid disease progression compared with those with slow disease progression [36]. In dengue infection, transcriptional signatures have enabled identification of differences in children with dengue virus fever from those who developed the more serious dengue haemorrhagic fever (DHF) [33,34]. Transcriptional differences could be seen in patients who subsequently developed DHF, prior to the development of the clinical features, including reduced expression of interferon response genes and differences in complement expression [33,34]. These studies have enabled the generation of hypotheses for further experiments to investigate the previously poorly understood aetiology of DHF, and in addition have identified potential early prognostic biomarkers [34].

Blood-derived gene expression signatures have also been seen to correlate with effective vaccination as measured by the quantity of neutralizing antibodies in yellow fever, influenza and pneumococcal vaccines [9,19,50]. These studies have not only identified putative biomarkers for predicting successful vaccination, but also enabled the description of the temporal kinetics of the transcriptional response to vaccination. Furthermore, they have identified the difference in transcriptional response between the influenza and pneumococcal vaccines. The influenza vaccine induced a greater anti-viral response as seen by the induction of interferon genes, compared with the pneumococcal vaccine, which induced more of an inflammatory response [9].

**Figure 1.**
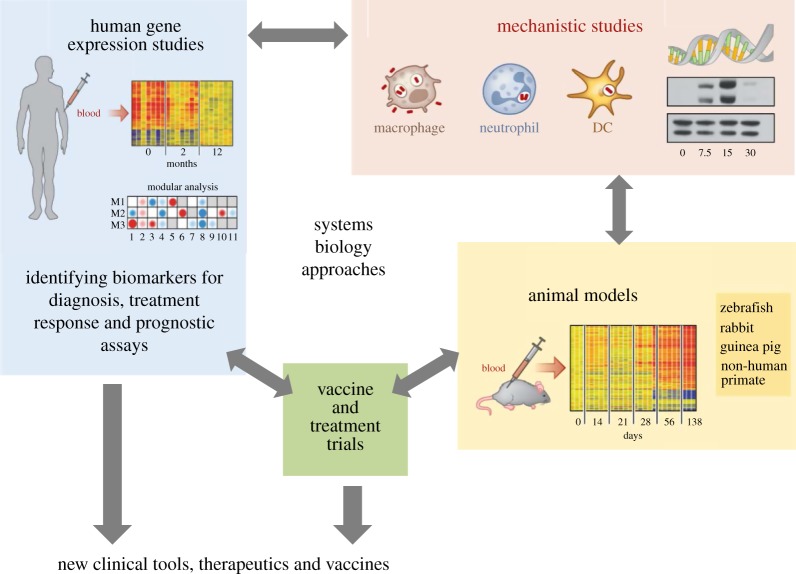
Using a systems biology approach in infectious disease research. Adapted from O‘Garra *et al*. [51].

Transcriptional signatures and systems biology approaches in infectious diseases can therefore feed into many different aspects of research, including translational clinical studies as well as basic immunological work (figure 1).

## 4. Tuberculosis: background of some of the current difficulties

*Mycobacterium tuberculosis,* the causative organism of tuberculosis, is estimated to have infected one-third of the world's population. In 2011, it was responsible for approximately 8.6 million cases of active tuberculosis and 1.4 million deaths [52]. *M. tuberculosis* is mainly spread via the aerosol route, and the outcome from inhalation of *M. tuberculosis* depends on a variety of factors: environmental and sociological factors, virulence of the *M. tuberculosis* strain and the capability of the host immune response [51]. The World Health Organization STOP TB strategy to halve the prevalence of tuberculosis by 2015 recognizes the need for new diagnostics, drugs and vaccines [53]. Development of these, however, is hampered by our incomplete understanding of the immune response to *M. tuberculosis* [51]. CD4^+^ T cells and the cytokines tumour necrosis factor, interleukin-12 and interferon (IFN)-γ have been shown to be critical in the control of *M. tuberculosis*, perturbations in these factors in animal models and humans being detrimental to the host [51]. These factors alone are not sufficient for an adequate host response however, and while numerous other factors have been identified, it remains unclear which combination of factors constitute a protective host immune response and what factors determine whether an individual goes on to develop tuberculosis [51].

Approximately 5–10% of those infected will develop active tuberculosis disease within the first year. The remaining 90% have clinically asymptomatic latent tuberculosis that carries an approximate 10% lifetime risk of progressing to active tuberculosis. Latent tuberculosis is defined as evidence of immunological exposure to *M. tuberculosis* with no symptoms of active clinical disease [54]. The host immune response is critical in maintaining this clinically latent state; perturbations in the immune system, either iatrogenic by immune modulating drugs or through diseases that compromise the immune system such as HIV infection, can lead to progression from latent to active tuberculosis [55,56].

In the non-HIV-infected individual, it is not possible currently to predict the outcome from *M. tuberculosis* infection. If it were, treatment of latent tuberculosis infection could be better targeted to those at the highest risk. Additionally, improved diagnostic tests for active tuberculosis would assist greatly in clinical management, enabling earlier diagnosis and hence prompt treatment initiation (which would also reduce the risk of onward transmission to others), as well as avoiding misdiagnosis of active tuberculosis and thus minimizing adverse events owing to unnecessary anti-tuberculosis treatment, which are common with current regimens [57].

Currently, there is only one licensed preventive vaccine against *M. tuberculosis*, the bacillus Calmette–Guérin (BCG) vaccine [58]. Its efficacy is not optimal and it has been shown to offer variable protection against the most common form of the disease—adult pulmonary tuberculosis [59–61]. A new, more effective vaccine is therefore required. Twelve vaccines are currently in phase 1 or phase 2 trials, although vaccine development is hampered by our poor understanding of what constitutes a protective immune response. The immunological readouts in current use do not provide correlates of a protective host response [62,63]. This may in part explain the inability of one of the most promising vaccine candidates to provide any addition protection above BCG vaccination in a phase 2 trial, despite demonstrating improved protection in animal models and induction of antigen-specific TH1 and TH17 cells in infants [64–66].

## 5. Tuberculosis peripheral blood gene expression studies

There has been a number of transcriptomic studies investigating the host response to *M. tuberculosis* infection; predominantly these have focused on the most common form of the disease—adult pulmonary tuberculosis (table 1).

**Table 1.** Summary of blood transcriptional profiling studies in tuberculosis. HC, healthy controls; LTB, latent tuberculosis; OD, other diseases; PBMC, peripheral blood mononuclear cells; TB, tuberculosis; TLR, Toll-like receptors. Modified from Berry *et al.* [67].

geographical region	year	sample	study design	key pathways	reference
South Africa, Malawi	2013	whole blood	TB versus OD TB versus LTB (HIV positive and negative)	—	[49]
UK	2013	whole blood	TB versus OD TB treatment	interferon signalling, role of pattern recognition receptors, antigen presentation	[16]
South Africa	2013	whole blood	TB treatment	complement; B-cell markers; CD64	[48]
Germany	2012	whole blood	TB versus OD	interferon signalling; complement; TLR signalling; Fcγ-receptor-mediated phagocytosis	[47]
South Africa	2012	whole blood	TB treatment	—	[25]
Indonesia	2012	PBMC	TB versus HC TB treatment	interferon signalling	[17]
The Gambia	2011	whole blood	TB versus LTB	JAK–STAT pathway; interferon signalling; TLR	[46]
USA and Brazil	2011	whole blood	TB versus LTB versus HC	interferon signalling	[45]
South Africa	2011	whole blood	TB versus LTB versus HC	—	[44]
UK and South Africa	2010	whole blood	TB versus LTB versus HC TB versus OD TB treatment	interferon signalling	[15]
South Africa	2007	whole blood	TB versus LTB	—	[43]
Germany	2007	PBMC	TB versus LTB	—	[42]

Several studies have been designed to attempt to identify a transcriptional signature that can differentiate active pulmonary disease from latent infection and/or healthy controls. Differentiating active tuberculosis disease from latently infected and healthy controls has enabled the identification of many immunological pathways that may be relevant to the pathogenesis of active tuberculosis such as type 1 and type 2 interferon signalling, Toll-like receptor signalling and also T- and B-cell function gene expression [15,17,25,42,43,45–48].

**Figure 2.**
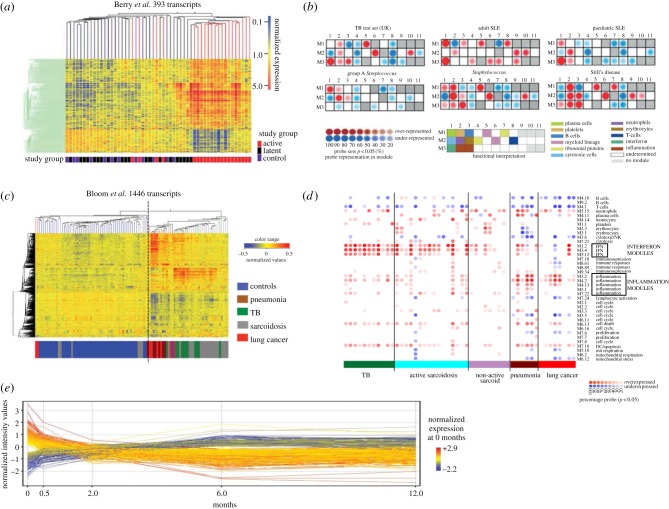
Transcriptional signatures in tuberculosis. (*a*) A 393 transcript signature was able to broadly distinguish active TB from latently infected and healthy controls (from Berry *et al.* [15]). (*b*) A modular approach is able to identify the key transcriptional differences between TB and other inflammatory diseases (modified from Berry *et al.* [15]). (*c*) A 1446 transcript signature reveals that pulmonary granulomatous diseases display similar transcriptional signatures that are distinct from pneumonia and lung cancer (modified from Bloom *et al.* [16]). (*d*) Modular approach showing the differences between active sarcoidosis, TB compared with pneumonias and lung cancer (modified from Bloom *et al.* [16]). (*e*) A 664 transcript signature is seen to change on treatment by as early as two weeks (modified from Bloom *et al.* [25]). SLE, systemic lupus erythematosus; TB, tuberculosis.

Early studies have identified interferon-inducible, inflammatory and chemokine genes as being differentially expressed between active pulmonary tuberculosis patients and controls. However, these studies involved small numbers of patients and had no independent test or validation sets [42,43]. In 2010, work involving patients from both the UK and South Africa identified a neutrophil-driven interferon signature present in active disease that was absent in the majority of healthy and latently infected individuals, and which correlated with the extent of radiographic lung disease and was diminished with anti-tuberculosis therapy (figure 2*a*). This transcriptional signature was validated with independent test and validation sets [15] and has subsequently been confirmed in studies involving patients from different countries (South Africa, USA, China, The Gambia, Germany and Indonesia) by other groups, using different microarray platforms and different microarray analytical approaches [17,45–47,68]. Type I interferon signalling had been previously largely underappreciated [69,70] and is now the focus of further work to determine the importance of this type I interferon signalling and how it influences the outcome following *M. tuberculosis* infection [51]. Approximately 10% of the latently infected individuals in the Berry *et al.* study [15] had a signature of active tuberculosis. It is unclear, at the present time, whether these individuals represent subclinical disease, incipient conversion from latent to active disease or another process. Further work is required to identify transcriptional signatures of latent *M. tuberculosis* infection that can predict those at risk of progression, and this will require longitudinal follow-up.

A meta-analysis of eight publicly available tuberculosis microarray datasets undertaken by Joosten *et al*. [71] found that after integration of the data from these separate studies interferon signalling was no longer a dominant pathway as had been described in many of the studies when the data had been analysed in isolation. Instead, there was enrichment for genes associated with myeloid cell inflammation, and TREM1 signalling was now found to be the most significant pathway [71].

To develop a diagnostic test based on gene expression levels, a transcriptional signature that can distinguish between active tuberculosis and other diseases is needed. An 86 transcript signature was able to differentiate between pulmonary tuberculosis and selected other diseases [15]. Using a modular approach, it was possible to identify distinct modular patterns for the different inflammatory diseases (group A *Streptococcus*, *Staphylococcus*, adult and paediatric systemic lupus erythematosus and Still's disease) and that tuberculosis had over-representation of interferon, inflammatory and myeloid lineage modules (figure 2*b*) [15]. However, further studies comparing tuberculosis with sarcoidosis as well as melioidosis revealed an overlap with the interferon-dominated pulmonary tuberculosis transcriptional signature [32,47,72]. These signatures have shown that diseases with similar pathophysiology may share similar transcriptional profiles—sarcoidosis, melioidosis and tuberculosis are all granulomatous diseases that can affect the lungs. However, when studies were designed that directly compared sarcoidosis and tuberculosis, blood transcriptional signatures could differentiate sarcoidosis from tuberculosis, and revealed an increased interferon-inducible response in tuberculosis in terms of the number of genes upregulated as well as in the magnitude of the response. Conversely, increased eukaryotic initiation factor 2 signalling was detected in patients with sarcoidosis [16]. One study revealed increased metabolic activity and antimicrobial defence response in tuberculosis when compared with sarcoidosis [47]. These studies demonstrate not only that diseases with similar pathological findings affecting the same organ can be distinguished using blood transcriptional signatures, but also that we can gain novel immunological insights from these blood transcriptional studies.

The specificity of any future diagnostic test based on transcriptional signatures will depend on its ability to accurately discriminate tuberculosis from a large number of different diseases. Importantly, it will be necessary to distinguish patients who are co-infected with HIV or not, as well as with comorbidities such as diabetes. Bloom *et al.* [16] set out to derive transcriptional signatures that could differentiate between active tuberculosis and other diseases (figure 2*c*). They included diseases that could clinically mimic tuberculosis, and by use of a modular approach to characterize the different diseases they again revealed the strong interferon response found in patients with active sarcoidosis and tuberculosis, but, in contrast, revealed an inflammatory nature of the blood transcriptome of patients with lung cancer or pneumonia (figure 2*d*) [16]. Previous studies had excluded HIV-infected participants in order to first define tuberculosis in the absence of any co-infection or co-morbidities. Additionally, there was concern that the reduced CD4+ T cells, as well an HIV transcriptional signature itself, would confound any potential analysis. More recently, Kaforou *et al.* [49] have published a large case–control study involving both HIV-infected and HIV-uninfected individuals. This study, by prospectively recruiting patients presenting with symptoms of TB who on further investigation were diagnosed with another disease, generated a large clinically relevant and diverse ‘other diseases’ cohort to act as a comparison group. In both their own datasets and an independent validation dataset, the derived transcriptional signatures were able to differentiate with a high degree of sensitivity and specificity between tuberculosis and healthy controls as well as tuberculosis and other diseases in both HIV-infected and HIV-uninfected individuals [49]. A transcriptional signature for use as a diagnostic will need to be able to diagnose TB from these real-world alternative diagnoses, and in addition the derivation of a signature that can distinguish patients in the context of HIV co-infection is of great importance as in several parts of the world a large number of tuberculosis cases are co-infected with HIV. This study is the first one to identify a transcriptional signature that is able to differentiate those that are *M. tuberculosis* infected from those non-infected despite HIV co-infection.

A transcriptional signature that reflects response to effective treatment has implications for the development of a clinical test that can determine a successful treatment response. Currently, sputum smear and culture testing after two months of therapy is the best predictor of relapse following treatment completion, but this is limited as sputum is not always accessible. A test that could identify treatment failure or success earlier would have implications for trials of novel drugs and regimens as well as aiding clinical practice by assisting in the identification of drug resistance or non-compliance earlier than is currently possible. Berry *et al.* [15] first demonstrated that significant transcriptional changes could be detected after two months following initiation of successful therapy. Two recently published studies following patients recruited from South Africa longitudinally showed that a transcriptional signature of active pulmonary tuberculosis quickly diminished with successful treatment [25,48]. Transcripts (1261) were seen to significantly fall in expression by one week. Among them were complement genes (C1q, C2 and derpin G1), which the authors hypothesized was related to the rapid reduction in mycobacterial load that was driving complement production [48]. A separate longitudinal study derived a 664 transcript signature that significantly changed over the course of treatment as well as by two weeks—this 664 transcript list was enriched for genes involved in interferon signalling [25].

## 6. Future

Gene expression microarray studies have advanced the immunological knowledge of the host response to infectious diseases, opening up avenues for further research as well as identification of potential biomarkers for use in diagnostic and prognostic tests as well as evaluations of response to treatment or vaccination.

RNA-Seq (also referred to as whole transcriptome shotgun sequencing) is an exciting developing new technology [1,73,74] that is starting to become available as a diagnostic approach to infectious and other diseases. RNA-Seq is a tag-based, high-throughput approach in which sequences are mapped against a reference genome thereby eliminating background signals and the need for statistical normalization. The technology has an advantage over microarray in that it additionally allows the detection of novel transcripts rather than reliance on known probe-based sequences. To date, the use of RNA-Seq for diagnosis in tuberculosis has been mostly restricted to looking at small sample numbers (in bovine tuberculosis [75]), and to the best of our knowledge, no reports are available for human tuberculosis. Currently, the cost, challenges of statistical data analysis and the logistics of large data storage currently make microarray a more practical option for analysis approaches that involve large samples/patient numbers. However, ongoing developments in next-generation sequencing, such as the recent United States Food and Drug Administration approval of the Illumina MiSeqD system for clinical use [76], make this a very exciting area for the future diagnosis of human disease.

Integration of the data from the complex host transcriptional signatures, host clinical phenotypes as well as pathogen genotype/gene expression using systems biology tools that continue to be developed and refined will enable researchers to better synthesize and understand the large volume of complex data being generated. This integrated analysis together with work in experimental models will provide a better understanding of the immunological response to infectious disease (figure 1). Gene expression data benefit from being widely available as an open resource for researchers. Many studies have already taken advantage of publicly available datasets to reanalyse data and integrate it to advance immunological knowledge [71,77] as well as to test or derive transcriptional signatures [16,30,32,47].

The identification of specific transcriptional responses for a disease will further our understanding of the host immune response and also aid in the development of specific tests that can be used in clinical practice. Further studies need to be undertaken to broaden the number of infectious diseases profiled, including co-infection studies, so that the transcriptional signatures may be better defined and be more applicable to ‘real-world’ situations. Furthermore, these transcriptional signatures will then need to be tested in large-scale prospective clinical trials to assess their ability to perform in large diverse populations. The ultimate use of transcriptionally derived signatures in the clinical management of infectious disease may not be as a standalone diagnostic, but rather used to support and aid in clinical diagnosis/management alongside other tools. For example, in active tuberculosis, a blood signature could support the current smear test, *M. tuberculosis* culture, clinical symptoms and imaging such as chest radiographs or cross-sectional imaging. For treatment response, it could be used alongside monitoring of clearance of *M. tuberculosis* from the sputum.
